# A rapid method for quantifying free and bound acetate based on alkylation and GC-MS analysis

**DOI:** 10.1186/s40170-016-0157-5

**Published:** 2016-09-02

**Authors:** Sergey Tumanov, Vinay Bulusu, Eyal Gottlieb, Jurre J. Kamphorst

**Affiliations:** 1 Cancer Metabolism Research Unit, Cancer Research UK Beatson Institute, Garscube Estate, Switchback Road, Bearsden, Glasgow, G61 1BD UK; 2 Institute of Cancer Sciences, University of Glasgow, Garscube Estate, Switchback Road, Glasgow, G61 1BD UK

**Keywords:** Acetate, ACSS2, Butyrate, Cancer metabolism, Formate, GC-MS, Histone acetylation, Stable isotope labeling

## Abstract

**Background:**

Acetyl-CoA is a key metabolic intermediate with roles in the production of energy and biomass, as well as in metabolic regulation. It was recently found that acetate is crucial for maintaining acetyl-CoA production in hypoxic cancer cells. However, the availability of free acetate in the tumor environment and how much tumor cells consume remains unknown. Similarly, much is still to be learned about changes in the dynamics and distribution of acetylation in response to tumor-relevant conditions. The analysis of acetate is non-trivial, and to help address these topics, we developed a rapid and robust method for the analysis of both free and bound acetate in biological samples.

**Results:**

We developed a sensitive and high-throughput method for the analysis of acetate based on alkylation to its propyl derivative and gas chromatography-mass spectrometry. The method facilitates simultaneous quantification of both ^12^C- and ^13^C-acetate, shows high reproducibility (< 10 % RSD), and has a wide linear range of quantification (2–2000 μM). We demonstrate the method’s utility by measuring free acetate uptake by cultured cancer cells and by quantifying total acetylation (using hydrolysis) in separate cellular compartments. Additionally, we measure free acetate in tissues and bio-fluids and show that there are considerable differences in acetate concentrations between organs in vivo, providing insights into its complex systemic metabolism and availability for various types of tumors.

**Conclusions:**

Our approach for the quantification of acetate is straightforward to implement using widely available equipment and reagents, and will aid in in-depth investigation of various aspects of acetate metabolism. It is also readily adaptable to the analysis of formate and short-chain fatty acids, making it highly relevant to the cancer metabolism community.

**Electronic supplementary material:**

The online version of this article (doi:10.1186/s40170-016-0157-5) contains supplementary material, which is available to authorized users.

## Background

Cancer cells have an incessant drive to proliferate, causing an unremitting demand for cellular building blocks to make new cells [1–4]. Among these building blocks are fatty acids that are incorporated into membrane lipids. Cancer cells acquire a significant proportion of their non-essential fatty acids through de novo synthesis [5–7]. The precursor for fatty acid synthesis is acetyl-CoA (AcCoA), of which the 2-carbon acetyl units are ligated to make the fatty acid palmitate, which in turn is subjected to elongation and desaturation reactions to generate a diversity of fatty acids.

AcCoA is located centrally in both catabolic and anabolic pathways. Therefore, it also acts as a rheostat of a cell’s metabolic state. This occurs in part by a direct effect of AcCoA on enzyme activity through allosteric regulation and other means [8, 9]. Additionally, enzyme activity is modulated by protein acetylation. Indeed, most enzymes of central carbon metabolism are acetylated to control activity [10]. Acetylation is especially prominent on histones and is important for controlling gene expression [11–13]. Based on this, it is not surprising that acetyl-transferases, sirtuins, and histone deacetylases have been found to play important roles in tumorigenesis [14, 15].

Because of its role in biomass production, cancer cells have a high demand for AcCoA. In nutrient and oxygen-replete conditions, this demand is largely met by mitochondrial AcCoA production from glucose carbon. This AcCoA is then used to make citrate, which in turn is transported to the cytosol to produce cytosolic AcCoA. In hypoxic conditions, however, most glucose is shunted towards lactate, and AcCoA production from glucose is diminished [16, 17]. The observation of a concomitant increase in citrate M^+5^ labeling from U-^13^C-glutamine has led to the finding that glutamine, through reductive carboxylation, is used to make AcCoA [16, 18]. However, the exact net carbon contribution is still debated [19]. Recently, a third substrate was found to sustain AcCoA synthesis, especially in hypoxic and highly glycolytic tumor cells: acetate [17, 20, 21]. AcCoA synthesis from acetate occurs through AcCoA synthetases (ACSS), and the nucleocytosolic ACSS2 was found to be important for maintaining tumor growth [20, 21].

As we discussed previously, while the fractional labeling of nucleocytosolic AcCoA from U-^13^C-acetate has been determined [17], this may not reflect the actual carbon contribution. The 2-carbon units from AcCoA could exchange back and forth with acetate, causing mixing of label that is not a representative of net flux. In extreme cases, significant labeling of AcCoA, and hence fatty acids, from acetate may be observed without a net carbon contribution [19]. Conversely, a potential exchange between intermediates from other substrates, like glutamine, would lead to an under-representation of the contribution from acetate. Therefore, an exact understanding of acetate’s importance as a carbon source requires direct analysis of acetate uptake by cells. Such a method could additionally be used for analysis of acetylation dynamics in cells, a subject that remains understudied.

Over the years, methods based on a number of principles have been developed for the analysis of acetate. An older method includes enrichment of acetate using a distillation-diffusion unit based on mixing the sample with anhydrous sodium sulfate and sulfuric acid and heating in the distillation bulb [22]. The then formed acetic acid is captured by water in the receiving tube and then measured using radioactivity (using ^14^C-acetate) [23] or flame ionization gas chromatography [24, 25]. Later on, enzymatic assays and NMR gained popularity. These approaches facilitate acetate quantitation but lack the combined strength of high sensitivity, the ability to use stable-isotopes to determine turnover, and short analysis times. The use of mass spectrometry is promising in this regard, but due to the small size of acetate, it depends on a reliable derivatization approach. Over the recent years, a number of papers addressing this have been published, based on silylation and alkylation [26–28].

Here, we present a GC-MS-based method that was fully optimized and validated for the accurate determination of both free and bound acetate. This method is based on alkylation of acetate to propyl-acetate. It is rapid in terms of sample preparation and analysis and was found to behave favorably in terms of accuracy, repeatability, and linear concentration range. It also minimizes background levels of acetate, a problem that can easily complicate the analysis of this metabolite and of longer chain fatty acids. We demonstrate the method’s utility by studying uptake of U-^13^C-acetate from the medium by cultured cancer cells. We also show that free acetate levels differ considerably between organs, in vivo, indicating that acetate availability for tumors depends on their anatomical location. In addition, we determine the bound acetate content of the cell and its sub-cellular fractions (i.e., nuclear bound acetate). Finally, we show that by using alternative alkylating agents, other compounds with clear relevance in cancer, like formate, can also be measured. Thus, the method described here will facilitate in-depth investigation into the role of acetate, and with slight modification, of other short-chain fatty acids in cancer.

## Methods

### Chemicals

Methyl chloroformate (MCF), methyl *tert*-butyl ether (MTBE), sodium hydroxide, concentrated hydrochloric acid, pyridine, 1-propanol, propyl-acetate, *N*-acetyl-l-aspartic acid (NAA), *N*-acetyl-l-cysteine (NAC), essential free fatty acid and globulin free bovine serum albumin (BSA), sodium acetate, sodium U-^13^C-acetate, and sodium ^2^H_3_-acetate, all of analytical grade, were from Sigma-Aldrich.

### Cell culture

A549 human lung carcinoma cells were from ATCC, were regularly tested for *Mycoplasma*, and were passaged in Dulbecco’s modified Eagle medium (DMEM; HyClone, GE Healthcare) containing 10 % fetal bovine serum (FBS; Gibco, Thermo Fisher Scientific) and split at 80 % confluence. Experiments were performed in DMEM with 10 mM glucose and 2 mM glutamine (experiment culture medium). For determining U-^13^C-acetate uptake, A549 cells were plated a day before the experiment in 6-well culture plates in medium with 10 % dialyzed FBS (HyClone, GE Healthcare). At 0 h, medium was replaced with experiment culture medium with same serum conditions and with sodium U-^13^C-acetate (0.5 mM). Samples were collected at multiple time points (0, 24, 48, and 72 h) and spun down at 2500*g* for 5 min to remove cell debris. To study the effect of histone deacetylase (HDAC) inhibitors, cells were incubated for 4 h with 50 μM panobinostat (Cayman Chemical). Cellular biomass was determined using packed cell volume (PCV) tubes (VoluPac, Sartorius). For hypoxia experiments, cells were cultured in pre-equilibrated medium in hypoxic glovebox (Whitley Scientific) maintained at 37 °C, 5 % CO_2_, and 1 % O_2_ a day before the experiment.

### Extraction of total acetate from cells

For quantification of bound acetate in various cellular fractions (i.e., nuclear and residual cellular fractions), we used a nuclear extraction kit (Merck Millipore) as per the vendor’s protocol. Acidic extraction of histones was performed as described previously [29]. For the fractionation procedures, the cells were washed with cold PBS and lysed with buffers provided by the kit, all containing 50 mM nicotinamide (Sigma) and 10 mM sodium butyrate (Sigma). The efficiency of fractionation was verified by western blot, using NuPage gels (Invitrogen, Life Technologies) and nitrocellulose membranes. Lysates for western blot were prepared in RIPA buffer (Pierce) with a protease inhibitor cocktail (Sigma). Tubulin (1:2500; Sigma, T5201) and TATA-binding protein (TBP; 1:2500; Abcam, ab63766) were used as cytosolic and nuclear markers, respectively. Histone fraction purity was confirmed by staining with Ponceau S (BioRad). Protein concentrations for the isolated cellular fractions were determined using Bradford Protein Assay Kit (Bio-Rad).

Extraction of total (free and bound) cellular acetate was performed by saponification of the cell pellet in sodium hydroxide. Cell pellets obtained by trypsinizing cells in 6-well plates were transferred to pre-chilled (ice temperature) microfuge tubes, centrifuged at 100*g* and 4 °C for 5 min and washed with ice cold PBS containing 50 mM nicotinamide and 10 mM sodium butyrate (2×), and finally centrifuged at 4 °C at 500*g* for 5 min. Bound acetate hydrolysis was performed by saponifying 50 μL of the extract through overnight incubation with 200 μL 10 M sodium hydroxide in a microfuge tube at 95 °C. Each sample was then cooled on ice before adding 150 μL of concentrated hydrochloric acid, followed by addition of 40 μL 1 mM internal standard sodium ^2^H_3_-acetate and drying by SpeedVac. The dried samples were reconstituted in 200 μL of water and further derivatized as below.

### Quantification of free acetate in tissues and bio-fluids

All animal work was performed in accordance with the European Directive 2010/63/EU and approved by ethical review process from the University of Glasgow. The heart, spleen, pancreas, kidney, liver, thymus, and lung tissues as well as urine and plasma were obtained from C57BL/6 mice (*n* = 7 for tissues and *n* = 5 for plasma and urine). Samples were snap-frozen on dry ice directly after collection, and tissues were ground using a mortar and pestle (both dry ice temperature). Aliquots of ground tissue (30–150 mg) were subsequently transferred to −20 °C Precellys lysing tubes (KT03961-1-003) containing 40 μL of 1 mM sodium ^2^H_3_-acetate and 200 μL methanol-water (1:1, *v*/*v*), for homogenization using a Precellys24 system (Precellys). The homogenates were then transferred into ice-chilled microfuge tubes, the lysing tubes were rinsed with 400 μL methanol-water (1:1, *v*/*v*) and fractions combined. The samples were centrifuged at maximum speed at −5 °C for 15 min, and supernatant was transferred to a new microfuge tube and dried by SpeedVac. Dried tissue extracts were resuspended in 200 μL water and derivatized as below. Mouse urine and plasma samples were directly subjected to derivatization using the procedure below. Human plasma samples were provided by Professor Iain McInnes, University of Glasgow, under the Ethics application 200150019 “The isolation of cells and soluble mediators from the blood of healthy volunteers”.

### Sample derivatization and analysis

#### Chemical derivatization of acetate

Note: because of the organic solvents and reagents, the derivatization should be performed in a fume hood. 200 μL of sample was added to a 2 mL microfuge tube, followed by addition of 40 μL of 1 mM internal standard sodium ^2^H_3_-acetate (unless this was already added), 50 μL of 1-propanol, and 50 μL of pyridine. The tube was then placed on ice for 5 min. 100 μL of 1 M sodium hydroxide was then added, immediately followed by 30 μL MCF and vigorous vortexing for 20 s. As gas builds up in the microfuge tube during the derivatization reaction, keep the lid closed with one finger and carefully open after vortexing to relieve pressure or keep the lid open during vortexing. After vortexing, 300 μL of MTBE was added, the sample vortexed for another 20 s, and centrifuged at 10,000*g* for 5 min. 200 μL microliters of the resulting upper layer was transferred to a GC vial for analysis.

#### Acetate quantification by GC-MS

The acetate samples were analyzed with an Agilent 7890B GC system coupled to a 7000 Triple Quadrupole GC-MS system. The column was Phenomenex ZB-1701 column (30 m × 0.25 mm × 0.25 μm), with an oven program as described in Table 1. Samples (2 μL) were injected using split mode (0.5 bar, 25 mL/min split flow). The column gas flow was held at 1.0 mL of He per min. The temperature of the inlet was 280 °C, the interface temperature 230 °C, and the quadrupole temperature 200 °C. The column was equilibrated for 2 min before each analysis. The mass spectrometer was operated in scan mode between 2.2 and 2.7 min with a mass range of 30–150 AMU at 1.47 scans/s. Agilent Mass Hunter B.06.00 software together with R-based MetabQ software were employed for automated data processing using peak heights of m/z 61, 63, and 64 ions used to quantify ^12^C, U-^13^C, and ^2^H_3_-acetate, respectively (the peak shapes were consistently highly symmetric, and using either peak area or peak heights gave equivalent results) [30]. Peak heights of ^12^C and U-^13^C acetate were compared to the ^2^H_3_-acetate peak height, and absolute concentrations were obtained from a calibration curve.

**Table 1:** GC temperature program for acetate analysis

Start temperature (°C)	Ramp (°C/min)	End temperature (°C)	Hold time (min)
45	–	45	0.8
45	25	60	0
60	50	190	0

## Results and discussion

### Method description

**Fig. 1:**
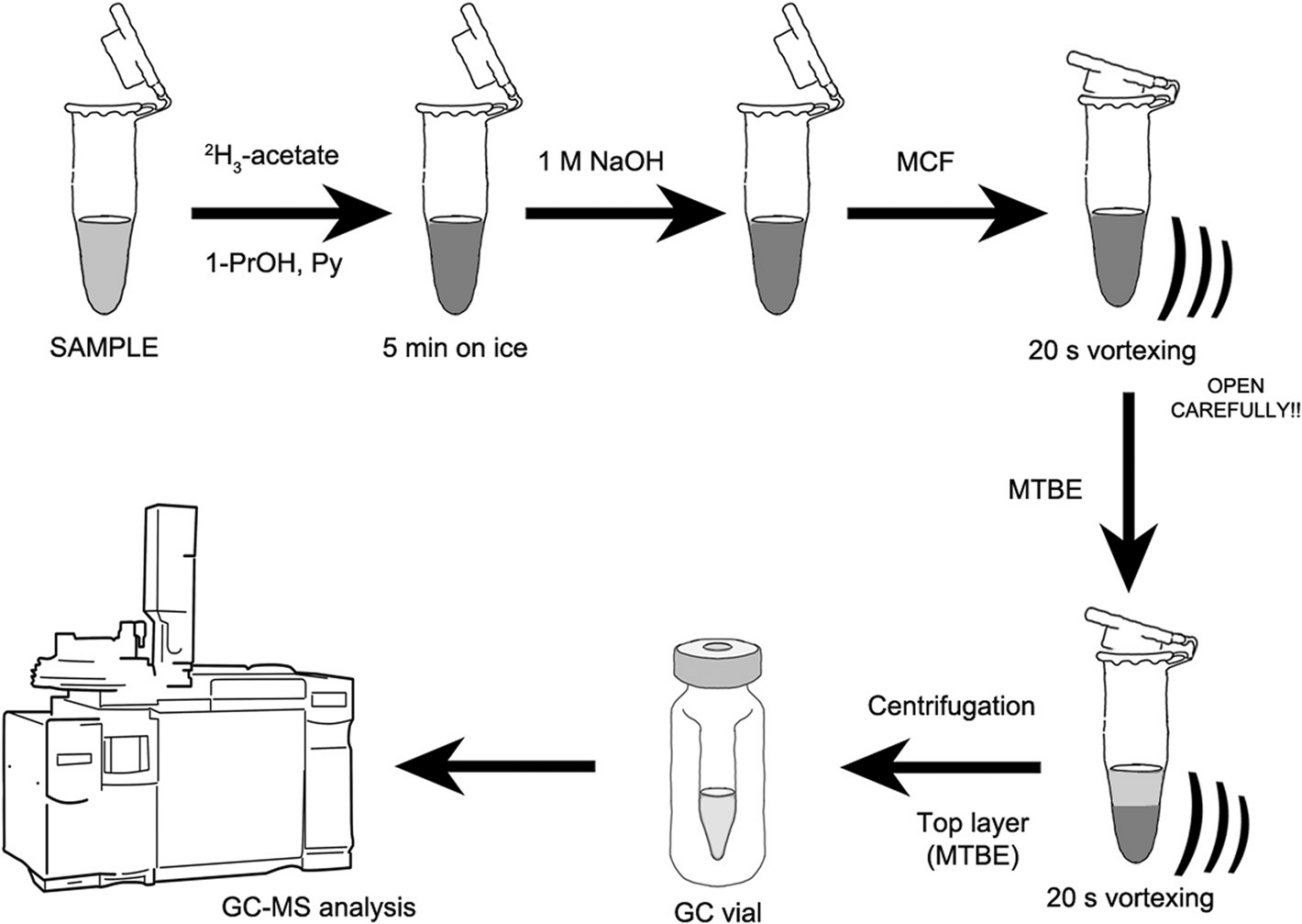
Schematic representation of the workflow for rapid acetate quantification. A sample aliquot was mixed with internal standard sodium ^2^H_3_-acetate, pyridine (Py), and 1-propanol (1-PrOH) followed by derivatization by adding sodium hydroxide and methyl chloroformate (MCF). After the derivatization, the sample was mixed with methyl tert-butyl ether (MTBE) and vortexed in order to extract acetate derivative (propyl-acetate). Thereafter, the sample was spun down and the top MTBE layer was transferred into GC vial for further GC-MS analysis

**Fig. 2:**
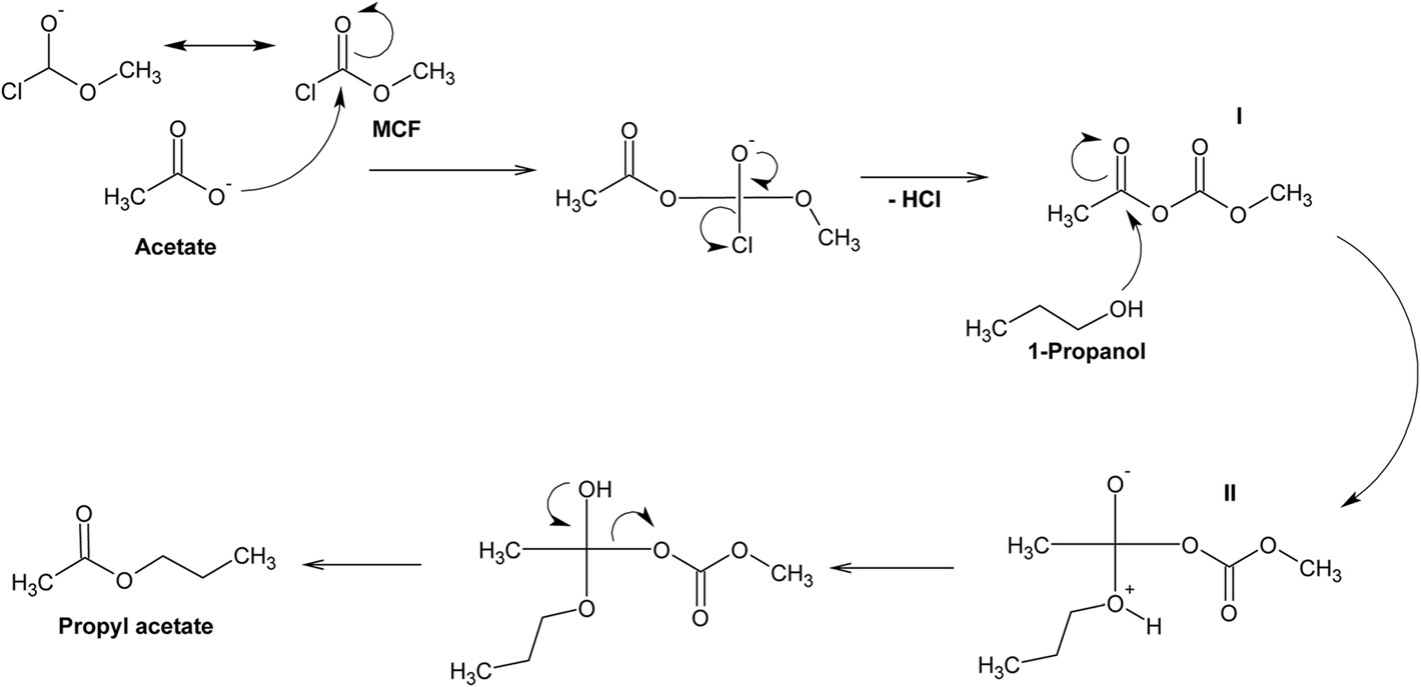
Chemical derivatization of acetate. Using methyl chloroformate (MCF), the carboxylic group of acetate is converted to a propyl ester: acetate first attacks MCF and the resulting intermediate (*I*) is then attacked by alcohol (1-propanol), generating a second intermediate (*II*), which undergoes further rearrangements to form propyl-acetate

We developed a robust and high-throughput method for absolute acetate quantification in biological samples. The method is based on derivatization using an established reaction with methyl chloroformate (MCF) [31, 32]. After sequentially adding internal standard, 1-propanol, pyridine, and sodium hydroxide, the derivatization reaction is initiated by adding MCF as soon as possible. The chemical modification occurs in basic conditions due to the presence of sodium hydroxide, while pyridine keeps the reaction system homogeneous as MCF does not dissolve in water alone (Fig. 1). 1-Propanol is used as a coupling reagent to produce propyl-acetate (Fig. 2). When sodium hydroxide is added to the cooled sample immediately prior to MCF addition, no hydrolysis occurs (see below). Alkylation of acetate with MCF facilitates rapid further modification of acetate in water-based conditions. In this case, the acetate-MCF intermediate (I) is attacked by the alcohol (1-propanol), and the resultant intermediate (II) undergoes further rearrangements, leading to the formation of propyl-acetate. Such derivatization approaches using the chloroformate family of reagents are well described [27, 31]. To quantify the yield of the derivatization and subsequent extraction into MTBE, we compared the MS signal intensity of 1 mM of acetate alkylated to propyl-acetate to an equimolar concentration of commercially obtained propyl-acetate in MTBE. Based on this, we found the recovery to be 95.5 ± 1.57 % (Additional file 1: Table S1).

The derivatization reaction with MCF is vigorous and exothermic, producing gases (hydrochloric gas, carbon dioxide) that cause pressure to increase within the tube. We therefore recommend following general health and safety rules when performing chemical derivatization, i.e., wear a lab coat, gloves, and protective glasses, while carrying out the reaction in a fume hood. To minimize the vigor of the reaction and hence pressurization, it is important to incubate the sample on ice (5 min) prior to derivatization. We recommend keeping the lid closed with one finger during vortexing and to carefully open the tube afterwards (a “popping” sound can be heard). Alternatively, the tube can be kept opened during the vortexing, depending on the researcher’s preference (we find that the tube’s contents do not spill when handled carefully). Afterwards, the derivatized acetate (propyl-acetate) can be easily extracted into organic solvent (MTBE) followed by the GC-MS analysis (Fig. 1).

**Fig. 3:**
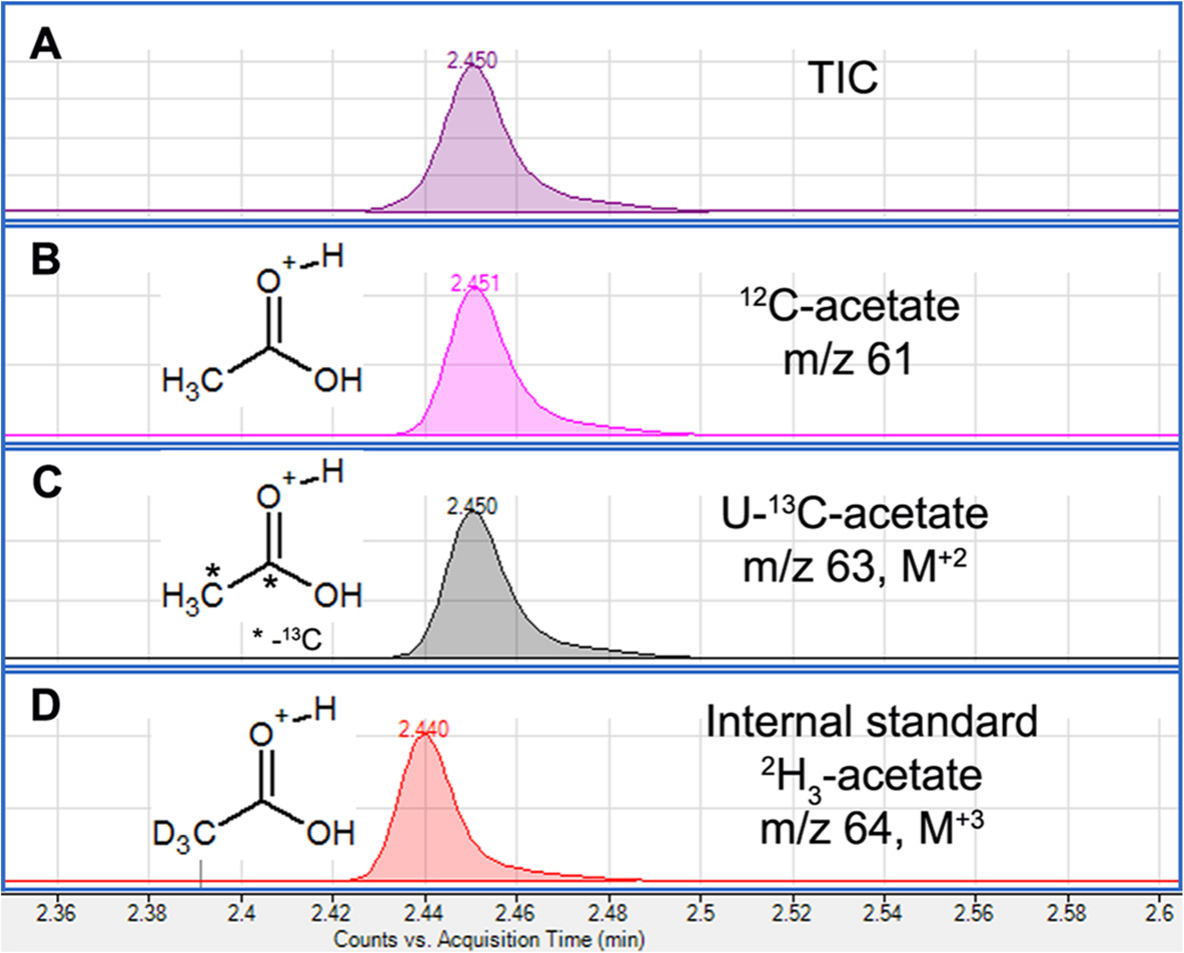
GC chromatogram for acetate derivative (propyl-acetate). **a** Total ion chromatogram. **b**–**d** Extracted ion chromatograms for ^12^C-, U-^13^C-, and ^2^H_3_-acetate, respectively. Ions m/z 61, 63, and 64 (structure of ions are indicated) were used for absolute quantification

Other primary alcohols (e.g., methanol and ethanol producing methyl and ethyl acetate, respectively) also can be used for derivatization. However, we found that with our mid-polar GC column (which we find to be very suitable for the analysis of a broad range of metabolites), propyl-acetate provides the optimal peak shape and retention time for rapid analysis. The sample preparation protocol described here is very rapid with a total derivatization time of a less than 1 min per sample. Together with the short GC-MS program with a total run time of 4 min per sample, this facilitates high-throughput analysis. While the acetate isotopologues ^12^C-acetate, U-^13^C-acetate, and ^2^H_3_-acetate have almost identical retention times, their peaks can be easily deconvoluted using specific ions (Fig. 3). As is generally observed in GC analysis of deuterated analytes, the ^2^H_3_-acetate derivative has a slightly shorter retention time due to inverse isotope effect [33]. Ions m/z 61, 63, and 64 for ^12^C_2_-acetate, U-^13^C_2_-acetate, and ^2^H_3_-acetate, respectively, were used for absolute quantification and showed optimal linear response.

### Method validation

To assess the repeatability of the sample preparation and analysis procedure, we determined the relative standard deviation (RSD) for ^12^C- and U-^13^C-acetate in freshly prepared standards at 50, 200, and 1000 μM for the respective acetate isotopologues. The method showed excellent repeatability with a RSD < 5 % for standards and < 10 % for biological samples (Additional file 1: Table S2). The linear range of quantification was assessed by analysis of serially diluted standards of both ^12^C- and U-^13^C-acetate within the range 2–2000 μM. We were unable to determine the limit of detection (LOD) for ^12^C-acetate as a background acetate signal of approximately 15 μM was always present in our samples, even though only reagents of the highest purity were used. It appears that acetate is a common trace contaminant in the atmosphere and reagents as well as in the plastic and glassware, similar to what has been observed for longer chain fatty acids [34, 35]. To investigate this further, we sought to determine the exact sources of background acetate (Additional file 1: Figure S1). As we analyze acetate in its derivatized propyl-acetate form, the background signal that we commonly observe could be either from propyl-acetate directly or from background acetate that is derivatized to propyl-acetate during the sample preparation. To look at the presence of background propyl-acetate, we performed an experiment in both plastic and glassware where we analyzed the level of propyl-acetate in reagents used for chemical derivatization. We observed that 1-propanol has the highest background propyl-acetate, but that it accounts for only ~10 % of the background acetate observed in procedure blanks (PB, i.e. blank samples that have been subjected to the entire sample preparation and mass spec analysis procedure), indicating that free acetate, rather than propyl-acetate is the major contaminant. Unfortunately, all the reagents are needed for the derivatization, so we are unable to determine what reagent contains the most background acetate. We did, however, perform the entire derivatization and extraction procedure on blank samples in both glass and plastic tubes. We found that in plastic, the resulting acetate background is ~50 % higher than in glass. Due to its convenience, we still prefer to work with plastic tubes and we leave this decision to the individual investigator.

Regardless of the background acetate, as the response proved highly linear, we were able to subtract the background signal (we quantify acetate background using blank samples that have been subjected to the entire sample preparation and mass spec analysis procedure, i.e., procedure blanks), resulting in a linear dynamic range from 2 to 2000 μM for both ^12^C-acetate and U-^13^C-acetate. The determined limit of quantification (LOQ) for U-^13^C-acetate was 0.1 μM. To keep ^12^C-acetate contamination to a minimum, we recommend preparing fresh reagents regularly (weekly), and we also recommend including procedure blanks routinely. Of note, we suggest users to quantify their own background acetate signal as it depends on the materials and reagents used and, therefore, it is very likely to be lab-specific.

We consider our approach to be an optimized alternative to other published derivatization methods, for the rapid measurement of acetate [26–28]. An approach based on silylation of acetate with *N-tert*-Butyldimethylsilyl-*N*-methyltrifluoroacetamide (MTBSTFA) showed a wide linear range for acetate quantification (0–3500 μM), high repeatability and low relative standard deviation (RSD < 5 %) [26]. It is, however, not suitable for processing water-based samples, the multiple extraction steps may lead to high background acetate levels, and the MS run time is long for analysis of acetate alone. An approach based on alkylation using propyl chloroformate (PCF) is similar to ours with respect to methodology and analytical performance but uses larger reagent volumes (which increases the risk for high acetate background levels) and longer MS run times as it was not optimized for acetate specifically, and it does not mention formate [27]. The published lower limit of quantification was comparable to our method (15 μM for current method versus 16 μM reported by Zheng et al. [27]). Zheng et al. [27] reported wider range of linear response, i.e., 16 μM–8 mM. We did not test beyond 2 mM as we have not observed such high levels in a physiological context. The reported repeatability was observed to be very similar with 0.54 % RSD (*n* = 6) reported by Zheng et al. [27], and ~0.7 % RSD (*n* = 3) depending on the acetate concentration, reported in current method for standard samples (relative standard deviations summarized in Additional file 1: Table S2). Finally, another GC-MS-based method for the analysis of short-chain fatty acids used 2,4-difluoroaniline and 1,3-dicyclocarbodiimide as condensation reagents, which necessitated 1-h incubation step [28]. Overall, the work presented here contributes the following, in addition to the already published methods [26–28]: (1) an optimized method for acetate as well as an adapted method to measure formate, (2) an in-depth discussion about background acetate, its potential sources, and how to deal with it in a practical manner, and (3) an approach to not only measure free acetate but also bound acetate.

**Fig. 4:**
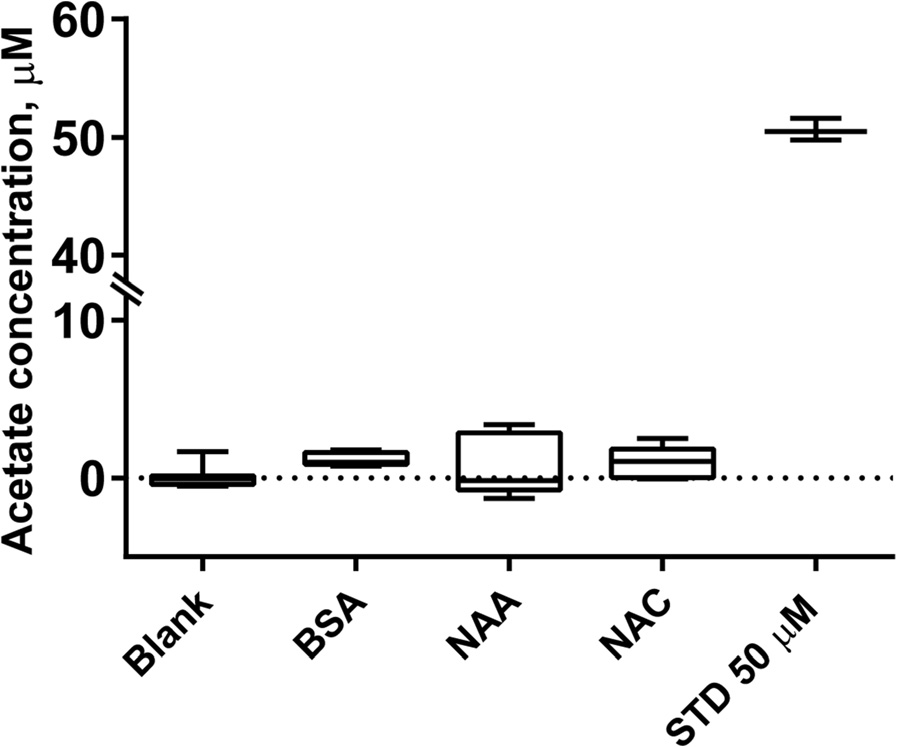
The effect of sodium hydroxide added during the derivatization on hydrolysis of bound acetate. *Whisker plot* for the relative acetate background derived from procedure blank and *N*-acetylated biomolecules—BSA, *N*-acetyl-l-aspartate (NAA) and *N*-acetyl-l-cysteine (NAC). No significant deacetylation occurs during the derivatization procedure. Data are means ± SD of *n* = 5 for all conditions. *Horizontal line* and *dot on box plot* correspond to median value and outlier, respectively. *Upper* and *lower end of box plot vertical line* are maximum and minimum determined values, respectively

The sample preparation protocol includes the addition of sodium hydroxide to facilitate the alkylation reaction. While this happens immediately prior to the derivatization reaction and the samples are cooled, this could potentially result in unwanted hydrolysis of acetylated bio-molecules, leading to increased levels of free acetate. To test if this occurs, we performed sample preparation and analysis on 10 mM solutions of *N*-acetyl-l-aspartic acid (NAA) and *N*-acetyl-l-cysteine (NAC), 1 g/L of BSA as well as procedure blanks. As amino acid acetylation is the most abundant source of bound acetate, and we did not observe a significant increase in signal, we conclude that the contribution from hydrolyzed bound acetate is negligible (Fig. 4).

### Acetate quantification in biological samples

Having established that our method can accurately and reproducibly quantify acetate, we next wanted to determine if it can reproduce previously established results. We therefore quantified acetate in plasma from eight healthy individuals. The plasma acetate concentrations varied from 20 to 51 μM with a median concentration of 34.1 ± 9.0 μM (data not shown). This falls within the previously published values of 41.9 ± 15.1 μM [36] and 30.4 ± 9.0 μM (Human Metabolome Database [37]).

**Fig. 5:**
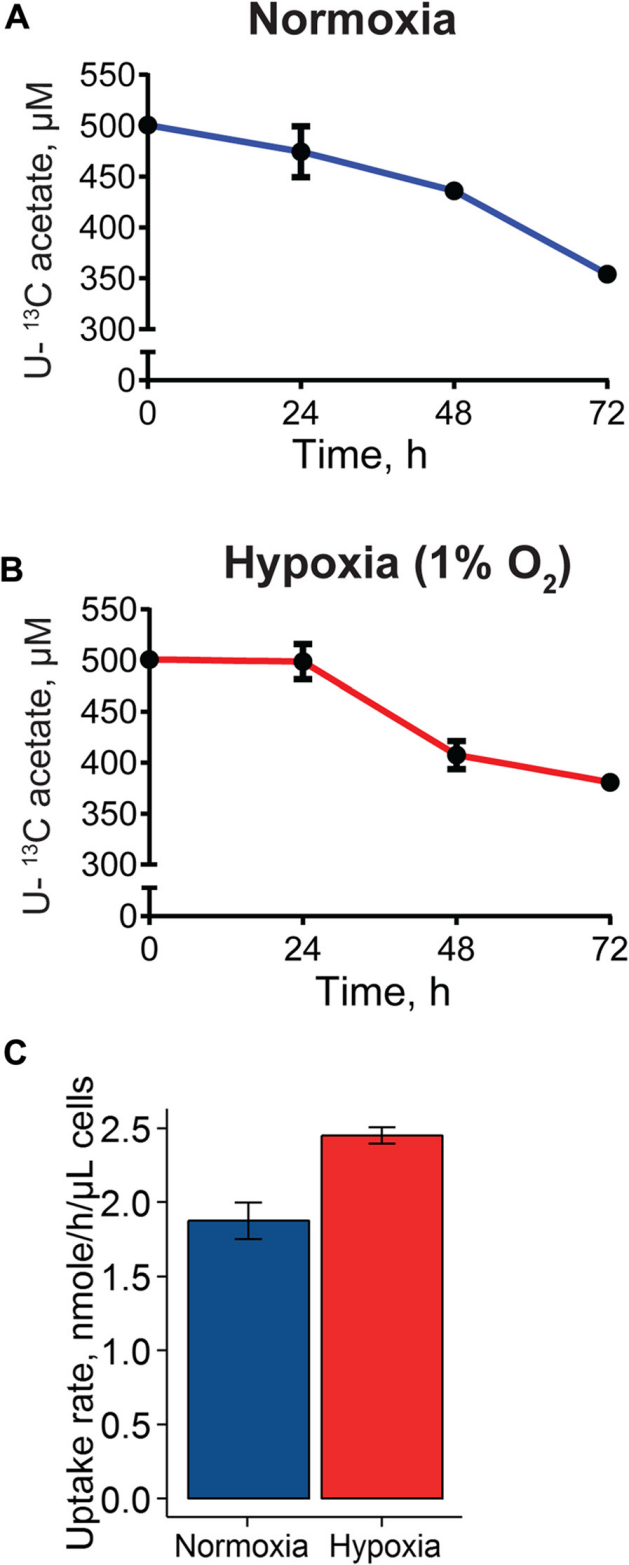
Acetate uptake by normoxic and hypoxic cancer cells. Concentration profile of U-^13^C-acetate in the medium of A549 cells in **a** normoxic and **b** hypoxic (1 % O_2_) conditions and **c** uptake rates calculated from (**a**, **b**). Values are mean ± SD (*n* = 3)

Recently, multiple studies identified an important role for the enzyme acetyl-CoA synthetase 2 (ACSS2) in mediating tumor growth during hypoxic and nutrient-limited conditions [20, 21, 38]. ACSS2 “activates” acetate to AcCoA so that it can be used for downstream metabolic reactions, including lipogenesis. Indeed, addition of U-^13^C-acetate to the medium of cultured cancer cells revealed increased labeling of lipogenic AcCoA, and consequently fatty acids, in hypoxic relative to normoxic conditions [17, 21]. However, to what extent this increased labeling is caused by an increase in acetate uptake remains unknown. To address this, we cultured A549 lung carcinoma cells in normoxic or hypoxic (1 % O_2_) conditions in medium containing 500 μM U-^13^C-acetate, and followed its concentration over time using our new method (Fig. 5). Consistent with the observed labeling of lipogenic AcCoA from U-^13^C-acetate, a robust consumption of U-^13^C-acetate by hypoxic cells was observed, as evidenced by a clear reduction in the medium. Surprisingly, while labeling of lipogenic AcCoA in normoxic conditions is considerably less than in hypoxia [17, 21], normoxic cells also displayed avid U-^13^C-acetate consumption. The acetate uptake rates for hypoxic cells were with 2.5 ± 0.1 nmole/h/μL cells (PCV) modestly higher than for normoxic cells (1.9 ± 0.1 nmole/h/μL cells). These results suggest that the increased labeling of lipogenic AcCoA from U-^13^C-acetate in hypoxia (~3-fold increase, data not shown) cannot be fully explained by increased acetate uptake. Instead, the increased labeling is likely partly caused by a drop in production of lipogenic AcCoA from glucose in hypoxic cells, leading to inflation of the relative contribution from acetate. We are in the process of investigating this further.

### Free acetate concentration in mouse tissues and fluids

**Fig. 6:**
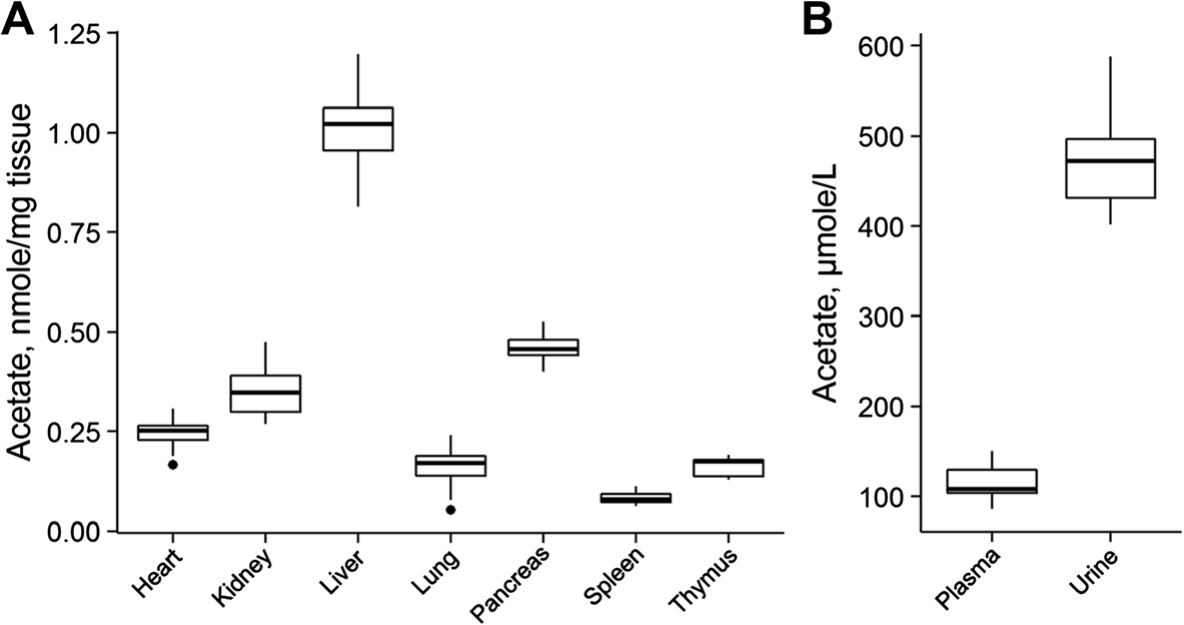
Concentration of free acetate in mouse samples. *Whisker plots* of (**a**) the heart, kidney, liver, lung, pancreas, spleen, and thymus tissues as well as for (**b**) plasma and urine were obtained from C57BL/6. Snap-frozen tissues were ground, and tissue aliquots were used for free acetate quantification. Data are means ± SD of tissue (*n* = 7) and fluid (*n* = 5) samples. *Horizontal line* and *dot on box plot* correspond to median value and outlier, respectively. *Upper* and *lower end of box plot vertical line* are maximum and minimum determined values, respectively. *Upper and lower horizontal lines* of a box are the 3^rd^ and the 1^st^ quartile, respectively

In vivo isotope tracing experiments demonstrated the utilization of exogenous acetate by tumors [20, 21], confirming a critical role for ACSS2 in mediating growth in various cancers [38]. However, the availability of acetate for solid tumors and how this varies between host organs remains largely unknown. In an attempt to address this, we analyzed tissues from multiple mouse (C57BL/6) organs (heart, kidney, liver, lung, pancreas, spleen, thymus) as well as plasma and urine (Fig. 6). Of all the organs analyzed, the liver contained the highest concentration of free acetate with an average concentration of 1.0 ± 0.1 nmole of acetate per mg of tissue (i.e., ~1 mM), more than twice as much as any other tissue. Nutrients absorbed by the intestine will first pass through the liver via the portal vein. High millimolar concentrations of acetate and other short-chain fatty acids have been reported to be generated by gut microbiota [39], and this provides a rationale for the high acetate concentration in the liver. As the concentration of acetate in the liver is much higher than in systemic circulation (i.e., plasma) and the lungs, which contain the first capillary system to be perfused by blood after liver passage, the liver appears to capture substantial amounts of acetate for metabolic use. Acetate was also considerably enriched in the pancreas and kidney relative to plasma, with concentrations of 0.5 ± 0.04 and 0.4 ± 0.06 nmole/mg (~0.5 and 0.4 mM), respectively. The reason for high acetate in the pancreas remains to be elucidated. One function of the kidneys is to clear water-soluble excess or toxic metabolites from the blood and considering that the acetate concentration in urine is substantially higher than in plasma, acetate may actually be actively excreted from the body as metabolic “waste”. Acetate levels were considerably less in other tissues with the lowest value found in the spleen at a concentration similar to plasma. Together, these observations highlight that the availability of acetate may vary considerably between organs, which has implications for tumor metabolism.

### Analysis of bound acetate in (sub)cellular and histone fractions

**Fig. 7:**
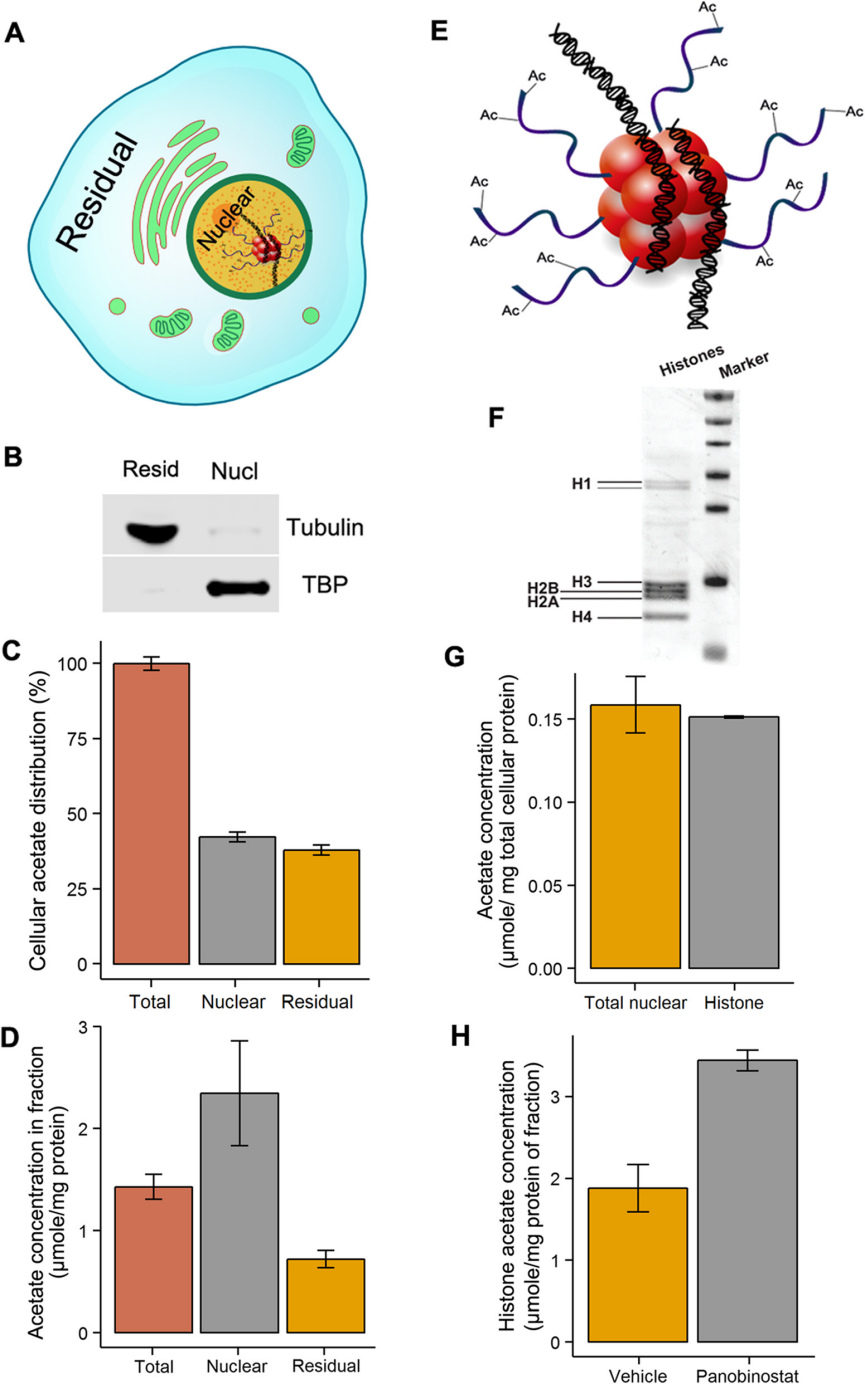
Quantification of (sub)cellular total acetate. **a** Schematic indicating what fractions were analyzed. **b** Western-blot showing quality of separation of the nuclear (TBP) and residual cellular fraction (tubulin). **c** Total acetate in whole-cell extract or nuclear and residual cellular fractions. **d** Same as (**c**), but expressed relative to protein amount in each fraction. **e** Histones (*red spheres*) are heavily acetylated and acetylation controls gene expression. **f** Western blot of histones following acidic extraction, stained with Ponceau S. **g** Amount of total acetate from acid extracted histones or nuclear fractionation. **h** Effect of HDAC inhibitor panobinostat on levels of acetate bound to histones. Values are mean ± SD (*n* = 3)

AcCoA occupies a central node in metabolism and is involved in biomass synthesis, catabolic pathways, and energy production. Because of this, AcCoA plays an important role in metabolic regulation [8]. This occurs in part through acetylation of bio-molecules, including a variety of proteins [2]. For example, acetylation of histones promotes gene transcription and a correlation between degree of histone acetylation and tumor aggressiveness has been observed [40]. Further evidence for the importance of acetylation homeostasis in cancer progression comes from the observation that disrupting acetylation dynamics by inhibiting deacetylases (i.e., HDACs, sirtuins) leads to potent induction of cancer cell death [12, 15]. While several aspects of acetylation are being extensively studied, much remains unknown about absolute pool sizes and turnover of acetate bound to bio-molecules in the various cell compartments, let alone how they are affected by tumor-relevant conditions. To address this, we combined our approach with hydrolysis in basic conditions. By heating samples and incubating them overnight with sodium hydroxide, ester bonds hydrolyze, releasing free acetate, which can then be derivatized and analyzed as before. Using this approach on a whole cell extract allowed us to quantify total (bound + free) acetate in A549 cells at 0.38 μmole/mg total cellular protein. We next asked if it would be possible to quantify bound acetate in separate cell compartments. We achieved a near-complete separation of the nuclear and residual cellular fractions (combination of cytosol and organelles other than the nucleus) using a commercial nuclear isolation kit (see “Methods” section) (Fig. 7b). We found that the bound acetate content was approximately equal for the nuclear and residual fractions (Fig. 7c). The sum of both fractions equaled ~80 % of the whole-cell measurement, which is most likely caused by reduced recovery during fractionation, but may also be caused by loss of free acetate. Expressing the acetate content per mg protein in each fraction revealed that the acetylation density in the nucleus is approximately threefold higher than in the residual cell fraction (Fig. 7d). Histones are known to be heavily acetylated, and to determine how much of the nuclear acetate was histone-bound, we compared the results of the nuclear isolation approach with a published acidic histone extraction protocol (Fig. 7e–g) [29]. Both approaches gave very similar values, indicating that nearly all bound acetate in the nuclear fraction is due to histone acetylation. Thus, histone acetylation alone accounts for half of the total cellular acetate.

This approach towards quantifying histone bound acetate may help to better understand the effect of histone acetylation in various cancer-related studies. To demonstrate the utility of the approach, we treated A549 cells with panobinostat, a pan-histone deacetylase (HDAC) inhibitor. A short, 4-h incubation with panobinostat caused a near doubling in the amount of histone-bound acetate, demonstrating that histone acetylation turnover is quite fast (Fig. 7h).

### Measuring formate and other short-chain fatty acids

By modifying the derivatization agent, the method is readily adaptable to other short-chain fatty acids, including formate. Coupling formate with benzyl alcohol generates formate derivative (benzyl formate), which can be readily analyzed by GC-MS (see supplemental experimental procedures Additional file 1: S1). We mentioned before that the GC-MS method for acetate analysis takes only 4 min, with mass detector operating between 2.2 and 2.7 min. The same method is able to analyze and quantify propionate and butyrate in the form of propyl-propionate and propyl-butyrate using the same sample derivatization method setup as for acetate. By extending mass detector operation time from 2.2 to 4 min, we were able to detect the propionate and butyrate peaks eluting at 2.92 and 3.30 min, respectively. Using ions m/z 75 and 89 for propionate and butyrate, respectively, it is possible to absolutely quantify these short-chain fatty acids.

## Conclusions

Here, we presented a robust and high-throughput method for absolute quantification of acetate using gas chromatography-mass spectrometry. It is based on a well-established chemical derivatization approach using methyl chloroformate and facilitates stable isotope tracing studies. In short, we anticipate that the analytical methods outlined here will be valuable to the cancer metabolism research community.

## Abbreviations

AcCoA, acetyl-CoA; ACSS, acetyl-CoA synthetase; AMU, atomic mass unit; ATCC, American type culture collection; BSA, bovine serum albumin; DMEM, Dulbecco’s modified Eagle medium; FBS, fetal bovine serum; GC-MS, gas chromatography-mass spectrometry; HDAC, histone deacetylase; LOD, limit of detection; LOQ, limit of quantification; MCF, methyl chloroformate; MTBE, methyl *tert*-butyl ether; MTBSTFA, *N-tert*-Butyldimethylsilyl-*N*-methyltrifluoroacetamide; NAA, *N*-acetyl-l-aspartic acid; NAC, *N*-acetyl-l-cysteine; NMR, nuclear magnetic resonance; PBS, phosphate buffered saline; PCF, propyl chloroformate; PCV, packed cell volume; RSD, relative standard deviation; TBP, TATA-binding protein

## Additional file

Additional file 1: Table S1. Derivatization efficiency of acetate. **Table S2.** Summary of relative standard deviations (RSD) for analyzed acetate samples. S1. Protocol for formate analysis and quantification. **Table S3.** GC temperature program for formate analysis. **Figure S1.** Assessment of background propyl-acetate and acetate levels. (DOCX 398kb)
