# Supplementary material for: A rapid method for quantifying free and bound acetate based on alkylation and GC-MS analysis
Source: Cancer Metab. 2016 Sep 2;4(1):17. doi: 10.1186/s40170-016-0157-5 (PMC5009658; doi:10.1186/s40170-016-0157-5)
Supplement: Additional file 1: Table S1. — Derivatization efficiency of acetate. Table S2. Summary of relative standard deviations (RSD) for analyzed acetate samples. S1. Protocol for formate analysis and quantification. Table S3. GC temperature program for formate analysis. Figure S1. Assessment of background propyl-acetate and acetate levels. (DOCX 398kb) [file 40170_2016_157_MOESM1_ESM.docx]

**Supplementary Information**

A rapid method for quantifying free and bound acetate based on alkylation and GC-MS analysis

Sergey Tumanov^1,2^, Vinay Bulusu^1,2^, Eyal Gottlieb^1^, and Jurre J. Kamphorst^1,2^*

^1^ Cancer Metabolism Research Unit, Cancer Research UK Beatson Institute, Garscube Estate, Switchback Road, Glasgow, G61 1BD, UK.

^2^ Institute of Cancer Sciences, University of Glasgow, Garscube Estate, Switchback Road, Glasgow, G61 1BD, UK.

*Corresponding author:

[Jurre.kamphorst@glasgow.ac.uk](mailto:Jurre.kamphorst@glasgow.ac.uk)

+44(0) 141 330 3966

Cancer Research UK Beatson Institute

Garscube Estate

Switchback Road

Bearsden, Glasgow

G61 1BD, United Kingdom

**Table S1. Derivatization and extraction efficiency of acetate.** To confirm the quantitative derivatization procedure yield we derivatized a standard mixture of acetate following MTBE extraction of produced propyl acetate. In parallel, we made an equimolar solution of commercially obtained propyl acetate analytical standard in MTBE that was directly subjected to GC/MS analysis. The moles of propyl acetate in standard and derivatized standard were equal. Peak intensities then were compared. Based on this experiment we found that the yield of propyl acetate from chemical derivatization and subsequent extraction was 95.5±1.57%.

| ***Replicate*** | ***Peak intensity of 1 mM***  ***acetate standard*** | ***Peak intensity of***  ***propyl acetate standard*** |
| --- | --- | --- |
| 1 | 15,938,297 | 16,692,960 |
| 2 | 16,154,060 | 16,579,144 |
| 3 | 15,858,054 | 16,912,599 |
|  |  |  |
| Mean | 15,983,470 | 16,728,234 |
| RSD, % | 0.78 | 0.83 |
| **Derivatization efficiency** |  | **0.955 (95.5%)** |

**Table S2. Summary of relative standard deviations (RSD) for acetate analysis in various sample types.**

| ***Sample*** | ***Replicates, n*** | ***RSD, %*** |
| --- | --- | --- |
| 50 µM acetate standard | 3 | 1.12 |
| 200 µM acetate standard | 3 | 0.74 |
| 1000 µM acetate standard | 3 | 0.76 |
| Mouse plasma | 5 | 6.05 |
| Mouse urine | 5 | 8.85 |
| Mouse heart | 7 | 4.23 |
| Mouse kidney | 7 | 7.49 |
| Mouse liver | 7 | 7.31 |
| Mouse lung | 7 | 9.03 |
| Mouse pancreas | 7 | 3.99 |
| Mouse spleen | 7 | 8.67 |
| Mouse thymus | 7 | 6.21 |
| Total cellular acetate | 3 | 8.57 |
| Bound histone acetate | 3 | 8.85 |
| Bound residual acetate | 3 | 8.66 |

**S1. Protocol for formate analysis and quantification**

***Chemical derivatization of formate***

40 µL of sample was added to a 1.5 mL microfuge tube, followed by 20 µL of 1 mM internal standard sodium ^13^C,^2^H-formate (M^+2^) (Sigma, CAS 1215684-17-5), 10 µL of benzyl alcohol (Sigma, CAS 100-51-6), 10 µL of 1 M sodium hydroxide and 50 µL of pyridine. The tube was then placed on ice for 5 min. Derivatization started by adding 20 µL MCF and vigorous vortexing for 20 seconds. Note: derivatization should be performed in a fume hood as gases are produced during the reaction. After vortexing, 200 µL of water and 100 µL of MTBE were added, the sample vortexed for another 20 seconds, and centrifuged at 10,000 *g* for 5 min. 50 µL of the resulting upper layer containing formate derivative (benzyl formate) was transferred to a GC vial for analysis.

***GC-MS analysis and formate quantification***

The same GC-MS instrument was employed for formate analysis as described for acetate analysis. Samples (2 µl) were injected into the GC-MS using split mode (0.5 bars, 25 mL/ min split flow). Gas flow through the column was held constant at 1.0 mL of He per min. The temperature of the inlet was 280 °C, the interface temperature 230 °C and the quadrupole temperature 200 °C. Reduced electron energy (60 eV) was used for analyte ionization. The column was equilibrated for 3 min before each analysis. The mass spectrometer was operated in SIM mode between 3.0 and 4.3 min with SIM masses of 136, 137 and 138 for M^0^, M^+1^ and M^+2^ (internal standard) formate, respectively. An oven program was as described in Table S1.

Total run time of a sample is 5.97 min.

**Table S3.** **GC temperature program for formate analysis.**

| Start temperature (°C) | Ramp (°C/min) | End temperature (°C) | Hold time (min) |
| --- | --- | --- | --- |
| 60 | - | 60 | 0.5 |
| 60 | 38 | 230 | 1 |

For data analysis Agilent Mass Hunter B.06.00 software was used, with peak heights of m/z 136, 137 and 138 ions used to quantify ^12^C, ^13^C and ^13^C, ^2^H_3_-formate, respectively. Identical retention time of 3.745 min was observed for all isotopologues. Peak heights of ^12^C and ^13^C -formate were normalized to ^13^C, ^2^H_3_-formate peak height and absolute concentrations were obtained from a calibration curve.

**
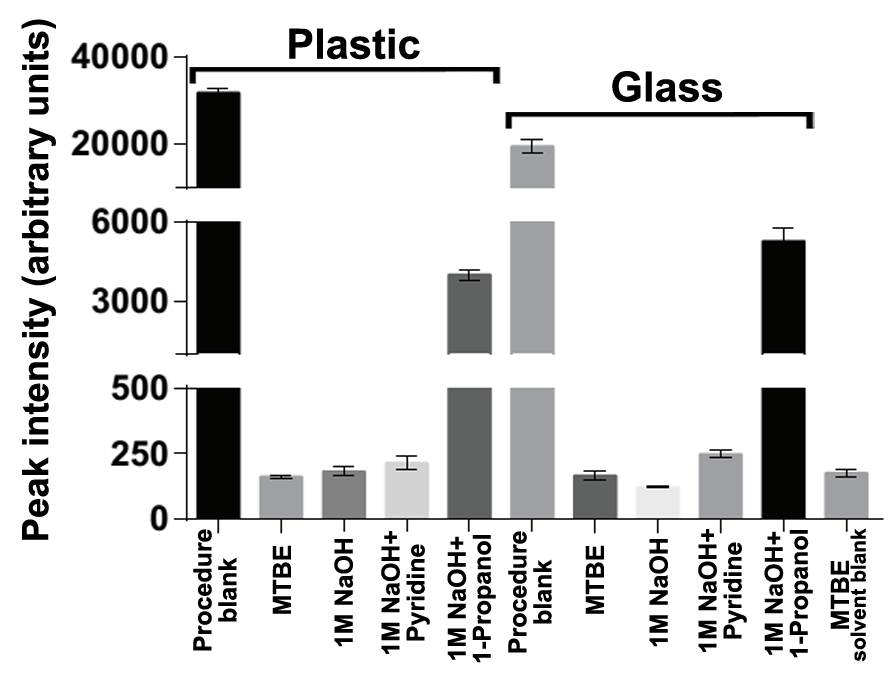
**

**Figure S1. Evaluation of sources of background acetate.** As we measure the propylated form of acetate (propyl-acetate) by GC-MS, the observed background can be from propyl-acetate directly, or from acetate that is derivatized to propyl-acetate. We determined background propyl-acetate in the various reagents by extraction into MTBE in either glass or plastic tubes. We also determined levels in MTBE exposed to plastic or glass tubes directly, and MTBE directly transferred to the (glass) autosampler vials (MTBE solvent blank). Measuring background acetate in individual reagents is not possible as all reagents together are needed to derivatize acetate. We did, however, perform the entire sample preparation procedure on blank samples, both in plastic and glass tubes (procedure blanks), to see if either added to the background acetate level.
